# E box motifs as mediators of proviral latency of human retroviruses

**DOI:** 10.1186/1742-4690-6-81

**Published:** 2009-09-16

**Authors:** Jean-Michel Terme, Sébastien Calvignac, Madeleine Duc Dodon, Louis Gazzolo, Albert Jordan

**Affiliations:** 1 Institut de Biologia Molecular Barcelona IBMB-CSIC, Parc Cientific de Barcelona, Baldiri Reixac 10, 08028 Barcelona, Spain; 2 Université de Lyon, UMR CNRS 5023, Laboratoire d'Ecologie des Hydrosystèmes Fluviaux, Campus de la Doua, 43, Boulevard du 11 novembre 1918, 69622 Villeurbanne Cedex, France; 3 INSERM U758 Virologie Humaine, IFR 128 Biosciences Lyon-Gerland, Ecole Normale Supérieure de Lyon, 4, Allée d'Italie, 69364 Lyon Cedex 07, France

## Abstract

The palindromic sequence motifs (CANNTG) known as E boxes are considered as binding sites for the basic helix-loop-helix (bHLH) class of DNA-binding proteins. Their presence has been reported in the long terminal repeats (LTR) of the HIV-1 and HTLV-1 proviruses. Their close proximity with the TATA region of both LTRs indicates that the bHLH proteins may act as important regulators of the function of proviral transcription. Indeed, observations on HIV-1 and recent results on HTLV-1 underline that these E boxes may be critically involved in the regulation of the proviral transcription of these human retroviruses. Indeed, of the two E boxes flanking the TATA sequences of the HIV-1 provirus, the 3' E box has been implicated in the transcriptional inhibition of viral gene expression. Such a role might also be played by the unique 5' E box present in the HTLV-1 LTR. In both cases, the expression of tissue-specfic bHLH proteins, like TAL1 might counteract the inhibitory effect exerted by E box proteins, thereby increasing proviral transcription. Finally, a phylogenetic study encompassing several subtypes of these two human retroviruses underlines that these E box motifs have recently appeared in the proviral LTRs and may be considered as potential mediators in the establishment of proviral latency.

## Introduction

The two prototypic human pathogenic retroviruses HIV-1 (Human Immunodeficiency Virus, type 1) and HTLV-1 (Human T cell Leukaemia Virus, type 1) infect their hosts on a long-term basis that relies on their abilities to infect latently specific cellular subsets, such as memory CD4^+ ^T cells. These viruses are thus able to efficiently escape the immune responses as well as the effects of anti-retroviral drugs that are included in highly efficient therapeutic protocols. Consequently, understanding the mechanisms that promote the establishment of latency is critical to the design of future therapies. A promising avenue of investigation is the search for host factors that would decrease proviral transcription. Hence, by considering data on HIV-1 and recent results on HTLV-1, we discuss lines of evidence showing that bHLH proteins may critically intervene in the proviral transcription of these two human retroviruses. Such an intervention is made possible through the presence of E-boxes in the long terminal repeats (LTRs) of the respective provirus.

## Discussion

The consensus hexanucleotide sequence known as the E-box motif (5'-CANNTG-3') represents the core DNA sequence capable of binding the basic helix-loop-helix (bHLH) class of proteins [1,2]. These proteins contain a basic DNA-binding region juxtaposed to the HLH domain that functions in protein dimerization. bHLH proteins include ubiquitous (class I, e.g. E47) and tissue-specific (class II, e.g. TAL1) transcription factors and play a prominent role in regulatory networks that control a diversity of processes from cell proliferation to cell differentiation. They can form homodimers or heterodimers with class II bHLH proteins and can act as transcriptional activators or repressors through the recruitment of distinct co-activator or co-repressor complexes, respectively [2,3]. Accordingly, the presence of E boxes in the LTRs of HIV-1 and HTLV-1 underlines the possible intervention of bHLH proteins in regulating proviral transcription.

### E box motifs in the LTR of human retroviruses

Indeed, four E box motifs have been described in the LTR of HIV-1; two are located 11 base pairs (bp) upstream and 6 bp downstream of the TATA sequence [4,5]. These two palindromic sequence motifs (CAGATG and CAGCTG) have been referred to as the 5' E box and the 3' E box, respectively [4]. More recently, the presence of a unique E box sequence (CATATG) in the LTR of HTLV-1, located 28 bp upstream the TATA box, has been reported [6]. The close proximity of these E boxes to the TATA region appears to be specific to the LTRs of HIV-1 and HTLV-1. In the 5' LTR of bovine leukemia virus, (an HTLV-1 related retrovirus), three E box motifs overlapping the cyclic AMP responsive elements (CREs) have been shown to be involved in transcriptional repression of BLV basal gene expression [7].

Disruptive mutagenesis experiments have underlined the functional importance of the TATA sequences and the flanking E boxes of the HIV-1 LTR, and more particularly of the 3' E box in regulating basal and Tat-induced gene expression [4]. Thus, it has been observed that one natural clone of HIV-1 carrying two mutated E boxes exhibited a high LTR basal activity in U937 cells [8]. More interestingly, experiments have been performed to characterize the bHLH proteins that bind to these E boxes in order to determine their role in the regulation of proviral transcription. Gel retardation analysis demonstrated that the specific binding of E box proteins (as either E47 homodimers or HEB homodimers or HEB/E47 heterodimers), and AP-4, a bHLH-ZIP protein, to the 3' E box of HIV-1 LTR [4,5]. Recently, the binding of AP-4 (and possibly other bHLH proteins) to the 3' E box was found to exclude the binding of TATA-binding proteins (TBP) to the TATA box and to inhibit the LTR-mediated transcription of the HIV-1 provirus *in vitro *[4,9]. Consequently, E boxes in the LTR may account for a modulation of viral replication, and even for the establishment and maintenance of latency in HIV-1 infected cells. The presence of an E box motif in the LTR of HTLV-1 and the observation that E47 was repressing both basal and Tax-induced LTR activity suggest that this bHLH protein indeed favors HTLV-1 proviral latency, possibly by interfering with the binding of TBP to the proximal TATA element [10]. The functional significance of the HTLV-1 putative E box has not been assessed yet. However, the above observations suggest that these E boxes are able to mediate the effect of bHLH proteins on the LTR activity of human retroviruses. Of interest, the overlap of E box elements and CREs in the BLV LTR has been proposed as a strategy to allow better silencing of viral transcription. In this model, suppression of viral gene expression has been shown to contribute not only to the impairment of immune surveillance, but also to the onset and progression of lymphoid tumours in BLV-infected sheep [11].

**Figure 1:**
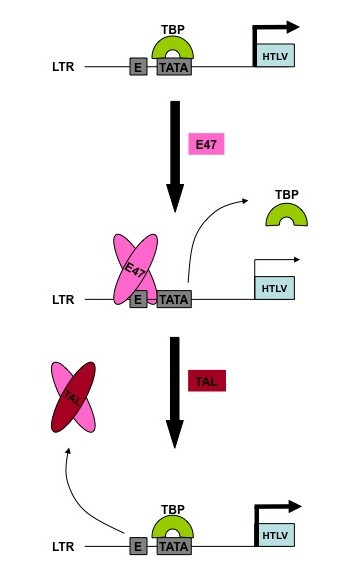
**Schematic representation of the proposed regulation of the HTLV LTR by E box proteins**. Binding of E box proteins to the adjacent E box disrupts the interaction of TBP with the TATA element and may inhibit basal expression thus favoring proviral latency. In turn, the expression of a bHLH class II protein (like TAL1) should interfere with the binding of bHLH factors to the E box, thus restoring the basal retroviral expression and turning off latency.

Collectively, these observations support that E box motifs in the LTRs of HIV-1 and HTLV-1 might represent important mediators of proviral transcription, by allowing the binding of bHLH proteins that might interfere with the transcriptional complex recruited at the TATA element. Consequently, some of these motifs would contribute to post-integration latency by turning off proviral expression. Furthermore, epigenetic mechanisms could also be implicated in the long-term suppression of viral expression, as E47 is known to bring chromatin-remodeling complex to specific promoters and therefore could induce epigenetic changes in proviral genome [12]. In return, that inhibition could be relieved by the action of tissue-specific class II bHLH proteins, such as TAL1 (T-cell Acute Leukemia 1), providing a way to exit proviral latency (Figure 1). Indeed, the binding of E47/HEB heterodimers to the HIV-1 3' E box is abrogated by the over-expression of a tissue-specific class II bHLH factor, TAL1, a functional inhibitor of E proteins [13]. Similarly, the over-expression of TAL1 is also able to counteract the E47 protein-mediated inhibition of the HTLV-1 LTR [10]. The regulation of proviral transcription during the early steps of T lymphocyte maturation might, therefore, be an important event contributing to the pathogenesis of this retroviral infection [14,15]. As an alternative to this scenario, one can imagine that the tissue-specificity of class II bHLH proteins may be linked to the differential ability of retroviruses to establish post-integration latency. For example, HIV-1 is known to establish latency in mature, but not in immature thymocytes [16]. Such a difference may be linked to the expression of TAL1 that is restricted to immature thymocytes. Clearly, much more experimental work is needed to assess the functional significance of these E boxes; however, the observations that have been devoted to delineate their intervention in the control of proviral latency are suggestive of their critical contributions.

### Evolutionary significance of E box motifs in the LTR of human retroviruses

As outlined above, the functionality of the E box elements needs further experimental evidence. Nevertheless, one can already address their evolutionary significance through the analysis of their distribution and conservation in LTRs of retroviruses that infect individuals, populations and species. Indeed such an analysis might provide valuable information on their involvement as mediators of proviral latency and on the evolutionary significance of the mechanisms highlighted above. At the scale of one to a few individuals, nearly all HIV-1 quasispecies exhibit intact E boxes (85 to 100% of intact E boxes) [8,17,18]. At a larger phylogenetic scale, E box mutants are strictly restricted to HIV-1 subgroups E, F and G (most virological studies have been performed with subtype B isolates) [19]. Interestingly, isolates of subtypes E and G while accounting for less than 9% of all HIV-1 infections were found to display a two- to three-fold higher LTR basal activity than those of subtype B, consistent with a role of the intact E box in decreasing viral expression [19,20]. Thus, the presence of intact E boxes in the HIV-1 LTR might account for a mechanism that regulates HIV-1 gene expression, as well as proviral latency.

**Figure 2:**
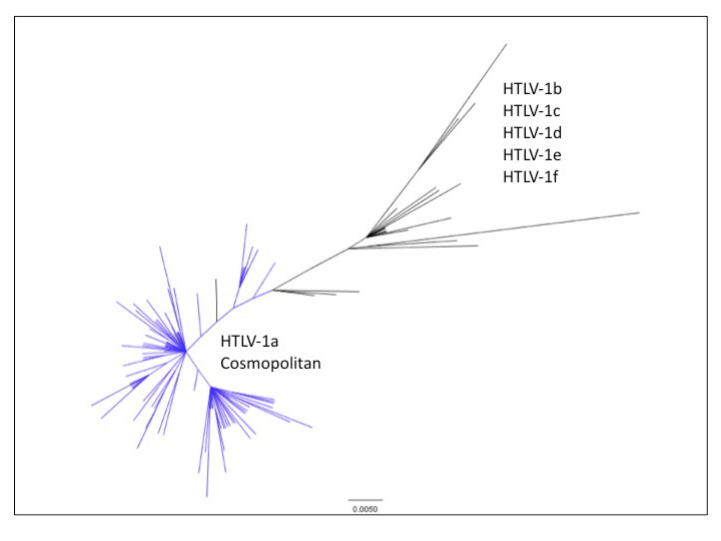
**Mapping putative E-boxes (CANNTG) onto a global HTLV-1 phylogeny**. HTLV-1 strains have been gathered into 6 groups of common ancestry (clades) named a to e, with HTLV-1a being often referred to as the Cosmopolitan clade [21]. This phylogenetic tree depicts the relationships of 127 strains (appearing as terminal, non-interior branches) among which >100 belong to the Cosmopolitan clade (the only pandemic HTLV-1 clade, solid line polygon). HTLV-1a is here largely overrepresented, which fits the reality of the pandemic; the other group distribution areas being much more restricted (*e.g*. Central Africa). Putative E-boxes have been mapped onto this phylogeny through the appending of a color code (blue = CANNTG consensus sequence; black = other sequences). This makes evident the presence of putative E-boxes in nearly all Cosmopolitan strains, which sharply contrasts with the situation prevailing in the other clades. This tree has been obtained through the analysis of a dataset derived from Coulthart *et al*.[22].

Considering that HTLV-1 replication occurs mainly by clonal expansion of infected cells (rather than by reverse transcription), this retrovirus is expected to lack intra-individual sequence variations. However, variations have been observed at the population level. On the basis of phylogenetic analyses, 6 groups of HTLV-1 (HTLV-1a to f) have been identified [21]. As the early diverged HTLV-1c sequences lack the putative E box [6], the ancestral state of HTLV-1 should be characterized by the absence of a TATA-neighbouring putative E box thus arguing for an active proviral transcription and reverse transcription. Of note, this putative E box (CANNTG) can only be found in the LTR sequences of HTLV-1a and HTLV-1e (as deduced from the analysis of alignments [6]). HTLV-1a, often referred to as the Cosmopolitan clade, represents by far the most widely distributed strain in HTLV-1 infected persons. It is striking to observe that an overwhelming majority (97%) of HTLV-1a isolates harbour this putative E box (as deduced from the analysis of alignments [22]), suggesting a selection for this motif during evolution (Figure 2). Such an observation might suggest that the presence of an E box might have contributed to the spread of the retrovirus, possibly by favouring the transcriptional repression of viral genes and thus facilitating virus- escape from the host immune system. Indeed, such a strategy might be critical to proviral maintenance via clonal expansion which necessarily precedes leukemia development.

## Conclusion

Even if HIV-1 and HTLV-1 have distinct characteristics and strategies to survive in the infected T cells, the capacities of these two human retroviruses to establish a latent infection is considered to be crucial for their pathogenesis. HIV-1 latency has been described to occur at both pre-integration and post-integration levels in infected CD4+ T lymphocytes [23-25]. More specifically, the presence of E box motifs in the HIV-1 LTR has been reported several years ago and raised their implication in the transcriptional regulation of the provirus. The E boxes in the LTR of HIV-1 might represent the binding sites of positively- and negatively-acting bHLH factors that arbitrate between episodes of active viral transcription and silenced gene expression [4]. Under these conditions, it is expected that bursts of viral replication will expose infected cells to the host's immune response, and lead to the gradual depletion of the CD4+ T-cell compartment, favoring immunodeficiency. Concerning HTLV-1, the primary infection is considered as a two-step process involving a transient step of reverse transcription followed by the Tax-induced polyclonal proliferation of infected cells, during genetic instability occurs [26]. Consequently, its is possible that E box proteins (and particularly E47) could contribute to decreased and silenced proviral transcription, that may favor the selection of a restricted number of latently-infected clones that escape the immune response and survive the genomic insults. Thus, proviral silencing might significantly contribute to the initiation of the leukemogenic process, during which the emergence of epigenetic events, such as LTR methylation [27,28], would in turn favor the long-term suppression of LTR activity. Finally and more importantly, evolutionary considerations also point out the potential importance of these E box motifs in the LTRs of HIV-1 and HTLV-1. Clearly, they plead for further functional investigation of the mechanisms involved in the recruitment of bHLH proteins to the E boxes and of the importance of these motifs as mediators of proviral latency.

## Competing interests

The authors declare that they have no competing interests.

## Authors' contributions

All authors read and approved the final manuscript.
